# Prenatal detection of terminal 9p24.3 microduplication encompassing *DOCK8* gene: A variant of likely benign

**DOI:** 10.1097/MD.0000000000023967

**Published:** 2021-01-22

**Authors:** Fagui Yue, Yang Yu, Xinyue Zhang, Yuting Jiang, Leilei Li, Ruizhi Liu, Hongguo Zhang

**Affiliations:** a Center for Reproductive Medicine, Center for Prenatal Diagnosis, First Hospital; b Jilin Engineering Research Center for Reproductive Medicine and Genetics, Jilin University, Changchun 130021, China.

**Keywords:** 9p24.3 microduplication, genetic testing, likely benign, prenatal diagnosis

## Abstract

Trisomy 9p is one of the most common chromosomal partial trisomies in newborns. However, reports on prenatal 9p microduplications are rare in the clinic. This study aimed to examine the genotype–phenotype correlation and assess the clinical significance of 9p24.3 microduplication encompassing the *DOCK8* gene. Eight pregnant women underwent amniocentesis for cytogenetic and genetic testing for various indications for prenatal diagnosis from January 2019 to January 2020. Chromosomal karyotypic analysis was performed on G-band metaphases that were prepared from cultured amniotic fluid cells. Chromosomal microarray analysis was carried out to detect chromosomal copy number variations. We also performed a literature review on clinical data on similar 9p24.3 microduplications to determine the genotype–phenotype correlation. We detected 123–248-kb microduplications in the region of 9p24.3 (chr9: 208454–469022), involving part of or the entire *DOCK8* gene. The indications for prenatal diagnosis mainly focused on the risk of maternal serum screening for trisomy 21/18, advanced maternal age, and increased nuchal translucency. No evident structural abnormalities were observed for all fetuses, except for case 5 who presented with increased nuchal translucency in prenatal ultrasound findings. Follow-up of postnatal health was performed and showed no apparent abnormalities for cases 1 to 6 after birth. The parents of case 7 chose to terminate the pregnancy while the parents of case 8 chose to continue the pregnancy. We propose that 9p24.3 microduplications that encompass part of or the entire *DOCK8* gene are variants that might be benign. However, further large-scale studies are necessary to evaluate the clinical pathogenicity. For prenatal cases with 9p24.3 microduplication, postnatal health and growth should be followed up and assessed regularly from childhood to adulthood.

## 1 Introduction

Trisomy 9p is referred to as partial or complete duplication of the short arm in chromosome 9. Trisomy 9p is one of the most common chromosomal partial trisomies, followed by trisomies 21, 18, and 13 in newborns. Since trisomy 9p was initially described in the 1970s,^[1]^ more than 150 cases of trisomy 9p have been reported in the clinic. Most trisomy 9p cases result from parental reciprocal translocation between chromosome 9 and another autosome, and only a limited number of these cases have de novo duplications.^[2,3]^ Patients with 9p duplication usually exhibit a wide spectrum of clinical manifestations, including growth and mental retardation, craniofacial dysmorphisms, limb/skeletal malformations, and kidney disorders.^[4,5]^

The genotype–phenotype correlation mainly depends on the duplicated size and genes involved in chromosomal segments. Chromosome 9p is recognized as a relatively poor gene region and compatible with survival.^[6]^ Currently, the 9p22–9p24 region is regarded as the minimal critical region, which is responsible for clinical features of trisomy 9p.^[7]^ The 9pter-p11.2 locus has been proposed as a critical region for development of Dandy–Walker malformations.^[8]^ The 9p23-p24.3 region encompasses genes that are implicated in autism spectrum disorders.^[9]^

As one of the most commonly detected autosomal structural aberrations, most reports on trisomy 9p involved postnatal cases, while prenatal reports are scarce in the clinic. Frequent copy number variations (CNVs) observed in fetuses without structural anomalies include 1q21.1, 15q11.2, 16p13.11, 16p11.2, 17p12, 22q11.21, Xp21.1, and Xp22.3.^[10]^

This study aimed to investigate the genotype–phenotype of 9p24.3 duplication and assess its clinical pathogenicity in prenatal and postnatal diagnosis. Our findings will provide clinical reference for genetic counselling of prenatally detected 9p24.3 duplication.

## 2 Materials and methods

### 2.1 Subjects

From 2019 to 2020, prenatal amniotic fluid samples were obtained from 8 pregnant women who were diagnosed with pure 9p24.3 microduplications following different prenatal indications at the Center for Reproductive Medicine and the Center for Prenatal Diagnosis of the First Hospital of Jilin University. We then followed up the children of these pregnancies and determined their postnatal health. The study protocol was approved by the Ethics Committee of the First Hospital of Jilin University (No. 2019–294), and written informed consent was obtained from all couples. All experiments in our study, including cytogenetic analysis and molecular cytogenetics, were performed in accordance with relevant guidelines and standard protocols.

### 2.2 Cytogenetic analysis

Chromosomal analysis was performed on G-band metaphases, which were prepared from 10 ml of cultured amniotic fluid cells in accordance with standard protocols in our laboratory. Twenty metaphases were analyzed for all samples. The International System for Human Cytogenetic Nomenclature (ISCN 2016) was used to describe the karyotype.^[11]^

### 2.3 Chromosomal microarray analysis

Following written consent from all pregnant women, 10 ml of uncultured amniotic fluid cells were collected using amniocentesis. Genomic DNA was isolated using the Qiagen micro kit (Qiagen, Hilden, Germany) following the manufacturer's protocol. Procedures were then conducted through the CytoScan 750K array (Affymetrix, Santa Clara, CA) in accordance with the manufacturer's protocol and our previous study.^[12]^ The procedure included genomic DNA extraction, digestion, and ligation, polymerase chain reaction (PCR) amplification, PCR product purification, quantification and fragmentation, labeling, array hybridization, washing, and scanning. Thresholds for genome-wide screening were set at ≥200 kb for gains and ≥100 kb for losses. The detected CNVs were comprehensively estimated by comparing them with the published literature and the following public databases:

1. Database of Genomic Variants (http://dgv.tcag.ca/dgv/app/home),
2. DECIPHER (http://decipher.sanger.ac.uk/),
3. ISCA (https://www.iscaconsortium.org/),
4. ECARUCA (http://www.ecaruca.net), and
5. OMIM (http://www.ncbi.nlm.nih.gov/omim).

Genomic positions refer to the Human Genome February 2009 assembly (GRCh37/hg19).

### 2.4 Selection of 9q24.3 microduplication cases

The present study focused on cases of 9p24.3 microduplication associated with different indications for prenatal diagnosis. Using this selection criterion, a systematic literature search was conducted with PubMed (https://www.ncbi.nlm.nih.gov/pubmed/) using relevant terms, including 9p24.3 duplication or trisomy 9p24.3, and by searching the databases of DECIPHER and ISCA.

## 3 Results

From 2019 to 2020, a total of 8 cases of pure terminal 9p24.3 microduplications were initially detected by chromosomal microarray analysis (CMA). All cases shared similar 9p24.3 microduplications involving part of or the entire *DOCK8* gene (chr9: 208454–469022). The distribution of indications for prenatal diagnosis was as follows: risk of Down syndrome (4/8), advanced maternal age (2/8), risk of Edwards syndrome (1/8), and increased nuchal translucency (1/8). G-banding analysis showed that all cases presented with normal karyotypes. The cytogenetic, CMA, and clinical findings of these cases are shown in Table 1. Among the 8 fetuses, the smallest duplication was 123 kb, while the largest was 248 kb. Because the parents of all fetuses did not undergo CMA, we failed to determine whether these 9p24.3 duplications were de novo or inherited. Cases 2 and 6 shared the same duplicated locus, and had a high risk in the second trimester of screening maternal serum. No severe structural abnormalities were observed in any fetuses, except for case 5 who had increased nuchal translucency. The parents of case 7 chose to terminate the pregnancy by their own volition. The parents of case 8 chose to continue the pregnancy and no other abnormalities were observed in the fetus. We then carried out a follow-up on postnatal health for cases 1 to 6, mainly focusing on congenital defects, developmental retardation, body stature, craniofacial dysmorphisms, and skeletal anomalies. No apparent abnormalities were observed in these infants, but a long-term follow-up investigation is still necessary.

**Table 1:** Summary of the cytogenetic, CMA results, and clinical findings of our cases with 9p24.3 microduplication.

			Birth		At investigation									
Case#	Sex	Pregnancy history	weight(kg)	length (cm)	weight(kg)	length(cm)	age	Karyotype results	CMA results(hg19)	Duplicated size(kb)	Inheritance	Duplicated gene	Prenatal diagnosis indications/reason of study	Follow-up outcome
1	F	G2P2	3.6	50	9.5	73	10 m	46,XX	9p24.3(208454-360401) × 3	152	n.a.	part DOCK8	DS: 1/245	No apparent abnormalities
2	M	G1P0	3.68	51	10.5	71	9.5 m	46,XY	9p24.3 (208454–332437) × 3	123	n.a.	part DOCK8	ES:1/11	No apparent abnormalities
3	M	G2P1	3.2	50	9.5	70	8.5 m	46,XY	9p24.3 (208454–368030) × 3	159	n.a.	part DOCK8	DS:1/404	No apparent abnormalities
4	M	G2P1	3.8	51	9.5	70	5 m	46,XY	9p24.3 (246690–394023) × 3	147	n.a.	part DOCK8	advanced maternal age	No apparent abnormalities
5	M	G3P1	3.55	50	8.5	62	3 m	46,XY	9p24.3 (208454–454023) × 3	245	n.a.	part DOCK8	Increased NT	No apparent abnormalities
6	F	G2P1	3.5	50	5	60	1.5 m	46,XX	9p24.3 (208454–332437) × 3	123	n.a.	part DOCK8	DS:1/87	No apparent abnormalities
7	TOP	G6P1	n.a.	n.a.	n.a.	n.a.	n.a.	n.a.	9p24.3 (363678–469022) × 3	163	n.a.	part DOCK8	advanced maternal age	TOP
8	N.A.	G1P0	n.a.	n.a.	n.a.	n.a.	n.a.	46,XX	9p24.3 (208454–475009) × 3	248	n.a.	DOCK8; part KANK1	DS:1/176	ongoing pregnancy

## 4 Discussion

Chromosomal microscopic imbalances, microdeletion, and microduplication, are associated with a series of prenatal/postnatal disorders, such as developmental disability, mental retardation, and congenital anomalies.^[13]^ For prenatal samples with normal karyotypic results, these subchromosomal aberrations of clinical significance occur in approximately 1% of structurally normal pregnancies and can be detected by CMA.^[10]^ Common abnormalities of chromosome 9 mainly focus on 9p13.3-p13.1 interstitial deletion, 9q22.3 deletion, 9q31.1-q31.3 deletion, and 9q34.3 deletion.^[14]^ To the best of our knowledge, this is the first report to focus on the clinical pathogenicity of prenatally detected pure 9p24.3 microduplications in which no fetal structural abnormalities were observed.

Because trisomy 9p is an easily recognizable syndrome, typical clinical phenotypes of trisomy 9p have been well described and characterized. These phenotypes include mental/developmental retardation, microcephaly, micrognathia, low-set or malformed ears, downslanting/small palpebral fissures, downturned corners of the mouth, a bulbous nose, hypotonia, hypertelorism, hand–foot anomalies, congenital heart disease, genitourinary anomalies, and delayed bone age. Some rare features of trisomy 9p, such as hepatoblastoma, epilepsy, self-injured behavior, lupus erythematosus, and multiple intractable keloids, have also been reported.^[15,16]^ Because most patients with trisomy 9p usually carry monosomy of another chromosome, determining the phenotype–genotype correlation can be difficult.

**Figure 1:**
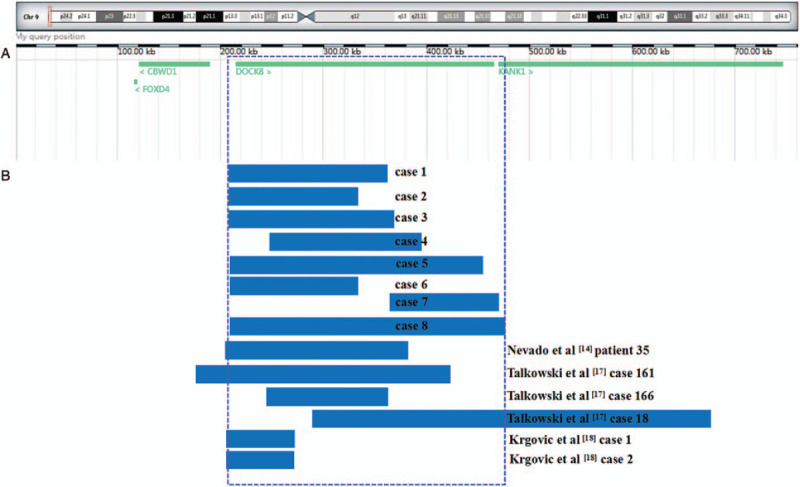
Scale representation of the duplicated region in the short arm of chromosome 9p24.3 (https://decipher.sanger.ac.uk/) (A) Location of genes in the region. (B) Microduplications detected in the present cases (cases 1–8) and previously reported similar microduplications in the region.

Patients with trisomy 9p, who are mainly postnatal cases, can usually be discovered through cytogenetic analysis owing to obvious chromosomal aberrations. However, 9p microduplications, which are scarcely reported in the clinic, can only be detected by molecular genetic techniques. To delineate the genotype–phenotype correlations of 9p24.3 duplication more clearly, we summarized clinical data involving similar duplications as in our cases (Table 2, Fig. 1).^[14,17–21]^ The duplications in all cases varied in size and ranged from 67 kb to 4.1 Mb and encompassed the 9p24.3 region. The age of the patients ranged from 10 months to 30 years. Among these cases, 4 patients (No. 1–4) were paternally inherited, 2 (No. 6 and 7) were de novo, and 6 (No. 5, 8–12) did not have available information. There was a high incidence rate of the following clinical characteristics: mental retardation (8/12), autism spectrum disorders (5/12), language/speech disorders (5/12), short stature (3/12), mood changes (2/12), and hand/foot anomalies (2/12). Additionally, epilepsy was discovered in 2 patients (No. 7 and 9) while 2 patients (No. 1 and 3) did not have epilepsy. All patients showed a wide range of clinical manifestations. The patterns of dysmorphic features were diverse, and showed normal phenotypes or varying degrees of anomalies. No obvious dysmorphic features were reported in 3 patients (No. 1, 3, and 10), and the father of patient 3 was unaffected. These findings suggest that partial 9p23p24.3 duplication is related to microcephaly, autism, and other clinical phenotype-related diseases.^[3]^ On the basis of the observations mentioned above, we consider that 9p24.3 duplication might be associated with mental retardation, autism spectrum disorders, and language disorder. The detected 9p24.3 microduplications in our cases appeared to be benign with an absence of any specific phenotypes. However, notably, all 9p24.3 duplications shown in Table 2 were postnatal cases, while our cases were prenatally detected with no fetal structural anomalies. Therefore, long-term follow-up studies are necessary to confirm whether there will be other clinical symptoms in the future in patients with 9p24.3 microduplications.

**Table 2:** Clinical features of previously published literature with similar microduplication at 9p24.3 with our cases.

No.	Sex/age	Duplicated region	Duplicated size	Inheritance	CMA results(hg19)	Critical genes	Clinical manifestation	References
1	F/n.a.	9p24.3	177 k	Maternal	9p24.3 (9:204090–381572) × 3	DOCK8	intellectual disability and/or congenital malformations	Nevado et al^[14]^ patient 35
2	M/n.r.	9p24.3	247kb	Maternal	9p24.3 (175632–422918) × 3	DOCK8, C9orf66	ASD, language delay, no dysmorphic features, significant hypotonia but walked at 18 m, normal neurological exam, no epilepsy, unavailable mother	Talkowski et al^[17]^ case 161
3	F/n.a.	9p24.3	118kb	Maternal	9p24.3 (243594–362341) × 3	DOCK8	ASD, normal IQ, verbal, unavailable mother	Talkowski et al^[17]^ case 166
4	F/n.a.	9p24.3	387kb	Paternal	9p24.3 (288719–676170) × 3	DOCK8, KANK1	ASD, low average nonverbal IQ, below average language, no epilepsy, no dysmorphic features, unaffected father	Talkowski et al^[17]^ case 18
5	F/15y	9p24.3	67kb	n.a.	9p24.3 (204193–271316) × 3	DOCK8	Palatoschisis, microcephaly at birth, discrepant intellectual profile, articulation problems/speech sound disorder, major depressive disorder, aggressive, impulsive, violent behaviour, non-suicidal self-injury	Krgovic et al ^[18]^ case 1
6	F/9y	9p24.3	67kb	*de novo*	9p24.3 (204221–271287) × 3	DOCK8	Intellectual disability at 3y, language disorder, ASD, aggressive, mood changes, tearfulness and stubbornness	Krgovic et al^[18]^ case 2
7	F/30y	9p24.2pter	4.1Mb	*de novo*	n.r.	DOCK8, DMRT1	Moderate MR, pachygyria, microcephaly, short stature, recurrent infections, epilepsy, retinal dysplasia, oedema, and facial dysmorphisms	Ruiter et al^[19]^ case 3
8	n.a.	9p24.3pter	0.1Mb	n.a.	n.r.	DOCK8,DMRT1	MR, Down syndrome-like dysmorphisms and behaviour	Ruiter et al^[19]^ case 4
9	n.a.	9p24.3	<0.2Mb	n.a.	n.r.	DMRT1,DOCK8	Learning problems, language disorder, and epilepsy	Ruiter et al^[19]^ case 7
10	M/9y	9p subtelomeric	1.0–2.1Mb	n.a.	n.r.	n.r.	Severe mental retardation, ASD and short stature but no obvious dysmorphic features, unknown parent.	Kok et al ^[20]^
11	M/10m	9pter	n.a.	n.a.	n.r.	n.r.	Prominent nasal bridge, small philtrum, bilateral rocker bottom feet, prominent ears, small eyes	Boggula et al^[21]^ no.3601
12	M/15y	9pter	n.a.	n.a.	n.r.	n.r.	Short stature, broad nasal bridge, prominent ears, 3^rd^-4^th^ cutaneous syndactyly and bilateral fifth finger chinodactyly	Boggula et al^[21]^ no. 901

Family members sharing the same 9p24.3 duplication show diverse phenotypes with varying degrees of severity, but the reasons for this finding are not yet fully understood. Variable expression and incomplete penetrance might explain why 9p24.3 duplications are occasionally transmitted through unaffected parents. The cumulative effects of variation in the DNA sequence across an individual's genome might also affect the phenotype.^[22]^ Additionally, the clinic phenotypes could also be a result of interactions between genetic variants and environmental factors. Furthermore, modifier genes that are located across the genome can alter the effect of another gene, which may play a role in the development of particular features and explain the variations and degrees of symptoms.^[23]^ Duplications might also cause diseases or specific features through triplosensitivity, gene disruption, or gene fusion at breakpoints.^[24]^

We also summarized comparable cases that harbored pure overlapped duplicated CNVs of 9p24.3 (chr9: 208454–469022) in the DECIPHER database (20 cases) and the ISCA database (43 cases). The proportions of pathogenicity were as follows: benign (21/63), uncertain (40/63), and pathogenic (2/63). These duplicated CNVs appeared to have low pathogenicity to some extent. Generally, establishing a well-described genotype–phenotype correlation for 9p24.3 microduplications in the future is optimistic. More evidence needs to be collected to elaborate on related features sharing a similar duplicated locus.

All CNVs that were detected in our cases encompassed part of or the entire *DOCK8* gene, and case 8 additionally carried part of the *KANK1* gene. The sensitivity of triplosensitivity for *DOCK8* and *KANK1* genes was not proven in the ClinGen database. Therefore, further delineation on the functions, implications, and potential effects of these genes would enhance the understanding on related clinical phenotypes. As a member of the DOCK180-related protein family, *DOCK8* (OMIM 611423), which contains 47 exons spanning 190 kb, is expressed in hematopoietic cells, brain tissue, the lungs, the pancreas, the kidney, and the placenta.^[25]^ Heterozygous mutations and deletions of *DOCK8* are associated with autosomal recessive hyper-IgE recurrent infection syndrome (OMIM 243700). *DOCK8* also plays a critical role in brain development and cognitive function, which are associated with mental or behavioral disorders.^[18]^ Heterozygous disruption of *DOCK8* due to chromosomal deletion or a translocation breakpoint is related to autosomal dominant mental retardation 2 (OMIM 614113).^[26]^ The effects of *DOCK8* duplication have not been clearly identified. According to clinical data in the general population of the Database of Genomic Variants, duplications involving *DOCK8* are not frequently present. These unaffected individuals made it ambiguous to attribute *DOCK8* duplication to carriers with clinical abnormalities, which suggests that *DOCK8* duplication is not causative. Previous studies on *DOCK8* duplications are limited and they indicate that rare CNVs might be benign for most patients who inherit from unaffected parents.^[19,25]^ However, Krgovic et al^[18]^ proposed that *DOCK8* duplications required investigation because of possible effects on clinical manifestations, and the gains of this gene should be at least regarded as a variant of unknown significance, not a benign CNV. On the basis of our observational study and the follow-up results, we propose that *DOCK8* duplication is likely to be benign. However, more studies and postnatal follow-up until adulthood for these cases should be carried out to support this speculation.

*KANK1* (OMIM 607704), which contains 18 exons, contributes to regulation of actin polymerization, actin stress fiber formation, and inhibition of cell migration via regulation of *RHOA* and *RAC1*. *KANK1* is ubiquitously expressed and plays critical roles in neurodevelopment and neurological function.^[27]^ Mutation or deletion of *KANK1* is associated with spastic cerebral palsy quadriplegic type 2 (OMIM 612900), which is a central nervous system development disorder that is associated with parent-of-origin-dependent inheritance. Affected individuals inherit deletion of *KANK1* from a paternal origin. Deletion of *KANK1* can result in imprinting-like inheritance. *KANK1* is maternally imprinted and deletion of the paternal copy could have clinical implications.^[28]^*KANK1* might also be related to autistic traits.^[16]^

There are some limitations to our study. Postnatal cases sharing similar 9p24.3 microduplications may present with diverse manifestations. Therefore, whether there will be delayed clinical symptoms in our cases should be clarified by further studies because our cases are currently young (cases 1–6) or unborn (case 8). Consequently, long-term follow-up of these cases’ postnatal health and growth is essential. Additionally, while the detected 9p24.3 microduplications in our study may not have been associated with structural abnormalities in prenatal ultrasonography, more relevant large-scale research is required to address this speculation.

## 5 Conclusion

We identified 8 prenatally detected terminal 9p24.3 microduplications cases, which encompassed part of or the entire *DOCK8* locus, with no structural ultrasound findings. We propose that the CNVs that were detected in our study were likely benign. However, further research on incomplete penetrance and variable expression of the *DOCK8* gene is still required. Although all of our cases are currently healthy, postnatal health and growth should still be followed up and assessed regularly from childhood to adulthood. Clinicians should offer comprehensive genetic counseling for such variations in prenatal cases to avoid causing anxiety for the couples. Parental CMA for identifying inheritance could be considered when necessary.

## Acknowledgments

We thank Ellen Knapp, PhD, from Liwen Bianji, Edanz Group China (www.liwenbianji.cn/ac), for editing the English text of a draft of this manuscript.

## Author contributions

**Conceptualization:** Hongguo Zhang.

**Data curation:** Yang Yu.

**Formal analysis:** Yang Yu, Xinyue Zhang, Yuting Jiang.

**Funding acquisition:** Fagui Yue, Ruizhi Liu.

**Investigation:** Xinyue Zhang.

**Methodology:** Yuting Jiang, Leilei Li.

**Software:** Leilei Li.

**Supervision:** Ruizhi Liu.

**Validation:** Ruizhi Liu.

**Writing – original draft:** Fagui Yue.

**Writing – review & editing:** Hongguo Zhang.
